# Development of a Premature Stop Codon-detection method based on a bacterial two-hybrid system

**DOI:** 10.1186/1472-6750-6-38

**Published:** 2006-09-02

**Authors:** Sebastián M Real, Diego M Marzese, Laura C Gomez, Luis S Mayorga, María Roqué

**Affiliations:** 1 Cellular and Molecular Laboratory -IHEM-, Faculty of Medical Sciences, National University of Cuyo, Mendoza, Argentina

## Abstract

**Background:**

The detection of Premature Stop Codons (PSCs) in human genes is very useful for the genetic diagnosis of different hereditary cancers, e.g. Familial Breast Cancer and Hereditary Non-Polyposis Colorectal Cancer (HNPCC). The products of these PSCs are truncated proteins, detectable in vitro by the Protein Truncation Test and in vivo by using the living translation machinery of yeast or bacteria. These living strategies are based on the construction of recombinant plasmids where the human sequence of interest is inserted upstream of a reporter gene. Although simple, these assays have their limitations. The yeast system requires extensive work to enhance its specificity, and the bacterial systems yield many false results due to translation re-initiation events occurring post PSCs. Our aim was to design a recombinant plasmid useful for detecting PSCs in human genes and resistant to bacterial translation re-initiation interferences.

**Results:**

A functional recombinant plasmid (pREAL) was designed based on a bacterial two-hybrid system. In our design, the in vivo translation of fused fragments of the *Bordetella pertussis *adenylate cyclase triggers the production of cAMP giving rise to a selectable bacterial phenotype. When a gene of interest is inserted between the two fragments, any PSC inhibits the enzymatic activity of the product, and translation re-initiation events post-PSC yield separated inactive fragments. We demonstrated that the system can accurately detect PSCs in human genes by inserting mutated fragments of the *brca*1 and *msh*2 gene. Western Blot assays revealed translation re-initiation events in all the tested colonies, implying that a simpler plasmid would not be resistant to this source of false negative results. The application of the system to a HNPCC family with a nonsense mutation in the *msh*2 gene correctly diagnosed wild type homozygous and heterozygous patients.

**Conclusion:**

The developed pREAL is applicable to the detection of PSCs in human genes related to different diseases and is resistant to translation re-initiation events. The diagnosis steps are easy, have a low cost, detect only pathologic mutations, and allow the analysis of separated alleles.

## Background

The presence of Premature Stop Codons (PSCs) in tumorsupressor and Mismatch Repair genes are a very frequent cause of hereditary cancer that account for 50%-90% of the reported pathogenic mutations [1-3]. PSCs are observed in the *apc *gene related to Familial Adenomatous Polyposis (FAP) [4,5], the *brca*1 and 2 genes related to Familial Breast Cancer [6-8] and the Mismatch Repair genes related to Hereditary Non-Polyposis Colorectal Cancer (HNPCC) [9,10]. PSCs are produced by nonsense or frameshift mutations resulting in the premature termination of proteins [11,12]. These truncated proteins can either loose completely their function producing haploinsufficiency in the cell or acquire a dominant negative effect on the full-length protein produced by the wild type allele [13,14]. In most cases, both consequences are pathologic. The mentioned genes related to hereditary cancers show an extremely heterogeneous mutation spectrum, but although the alterations are scattered throughout the complete coding sequence, most of the identified mutations produce premature termination of protein translation [15,16].

The detection of mutations in genomic DNA is one of the most common diagnosis methods used for these diseases – e.g. SSCP, DGGE, HA – [17,18]. These techniques have the advantage of using only DNA -an easy to handle molecule- and have widely proved their usefulness. Still, they have their limitations. They reveal all sequence changes, including silent mutations and polymorphisms without pinpointing the type of mutation. Therefore, subsequent analyses are sometimes needed to distinguish between pathologic mutations and polymorphisms [19].

The other major diagnosis methods are based on the detection of the protein product, and they have the advantage of detecting exclusively pathologic mutations. The translation machinery is exquisitely sensitive to PSCs that terminate the process of protein elongation. The analysis of the truncated proteins can be done in vitro – e.g. Protein Truncation Test (PTT) – by starting from either genomic DNA or RNA, amplifying the sequence to be analyzed, and using these products as templates for in vitro transcription and translation [20,21]. The shorter products of the mutated alleles are then distinguished from the full-length protein products of normal alleles. A disadvantage of the conventional PTT is the involvement of SDS-PAGE followed by autoradiography or Western blotting. As it relies on visual inspection to detect the mobility of shifted bands, it may also be subject to evaluator error. Another limitation of the method is the lack of sensitivity to diagnose mutations near the translation end that produce too small mobility shifts to be detected [20].

The PTT test is based on cell-free transcription and translation. However, the same machinery is present and functional in living cells. Therefore, in principle, it would be possible to carry out the diagnosis by using the translation machinery of cells. Several models have been developed in the past that use living-system strategies. For these methods, PCR amplifications of consecutive fragments of the gene are introduced in a reporter-plasmid, which is then transformed into yeast or bacteria [22]. The DNA to be tested is ligated upstream an easily detectable protein. The presence of a PSC in the sequence will stop translation prematurely and prevent the expression of the reporter protein.

The so called "yeast-based stop codon assay" has been reported for the detection of chain-terminating mutations in some specific genes. The PCR-amplified coding sequence is inserted by homologous recombination into a yeast expression vector system, and transformants are assayed for growing in a nutrition-deficient medium. To allow the insertion of the DNA sequence by homologous recombination, a gap-vector must be previously generated to introduce homologous flanking sequences for the posterior recombination insertion step. Therefore, this method requires the construction of an expression vector that is specific for each tested sequence (e.g. exon), and hence is not applicable to a new candidate gene. A modification made by Kataoka et al [23], consists of the development of a universal gap-vector to permit the automatic integration of any gene fragment. Each primer used to amplify the sequence of interest carries a 24bp recombination tail. However, this method requires performing a nested PCR to reduce nonspecific amplification products; hence, it has many steps for a routine diagnosis.

Bacteria have the advantage of being easier to manipulate than yeast. However, even though some authors have reported the development of bacterial systems based on the cloning of relevant segments in-frame with a colorimetric marker gene [24], no data was found reporting their application in routine diagnosis. A problem with this strategy is the capability of bacteria to re-initiate translation downstream of the STOP, causing expression of the reporter even in the presence of a PSC (false negative diagnosis) [25].

The aim of this work was to develop an alternative methodology to detect PSCs in human genes using the transcription and translation machinery of bacteria while avoiding the translation re-initiation interference. The strategy was to introduce the DNA to be tested in a linker between the two fragments of the catalytic domain of adenylate cyclase (cya) of *Bordetella pertussis*, used for the bacterial two-hybrid system developed by Karimova et al [26]. Re-initiation events will render two separate cya domains catalytically inactive. Our results show that the plasmid is a sensitive, specific, efficient and low-cost tool for the detection of PSCs and that it is insensitive to re-initiation artifacts.

## Results

### Development of the recombinant pREAL plasmid

**Figure 1:**
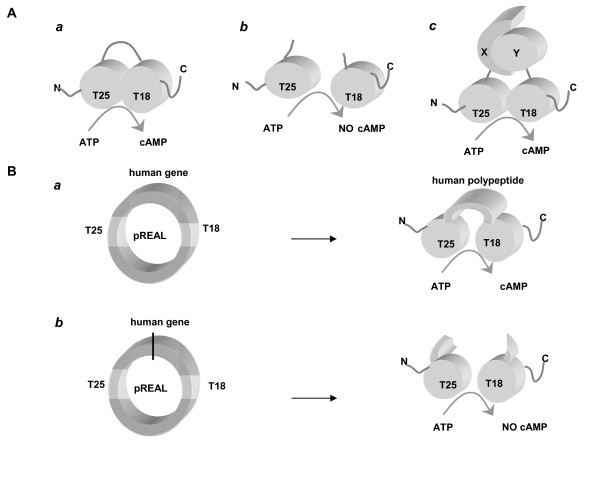
**Principles for the design of pREAL**. **A**: Principle of E.coli two-hybrid system based on functional complementation of adenylate cyclase domains of *Bordetella pertussis*. The two spheres represent the T25 and T18 domains corresponding to amino acids 1–224 and 225–339 of the cya protein. In *a*, the full-length catalytic domain, when expressed in the cya^- ^BTH102 strain exhibits a basal activity that results in cAMP synthesis. In *b*, the two independent fragments are unable to interact and no cAMP is synthesized. In *c*, the two fragments, fused to two interacting proteins -X and Y- are brought into close proximity, resulting in functional complementation followed by cAMP production. B: Principle of the design of the pREAL plasmid. Both adenylate cyclase fragments (T25 and T18) are subcloned into the same plasmid, leaving a multiple cloning site in between to insert a fragment of a human gene of interest. In *a*, T25 and T18 are linked by a human polypeptide chain and are functionally active when a PSC-free human gene is introduced in pREAL. In *b*, any PSC in the human gene inhibits the complete translation of both fragments as a single protein. In case of an in-frame translation re-initiation event post PSC, T18 will be present but unable to interact with T25; hence no cAMP will be synthesized.

Our aim was to develop an alternative methodology to detect PSCs in a human gene based on its fusion with a reporter gene and on bacterial phenotype screening. Previous developments in this field produced false-negative results, probably due to translation re-initiation events, so we designed a recombinant plasmid in order to avoid this artifact. The strategy was based on the bacterial two-hybrid system developed by Karimova et al [26] to identify interacting proteins. In essence, when the catalytic domain of *Bordetella pertussis *adenylate cyclase (encoded by the *cya *gene) is expressed in *E. coli*, it exhibits a strong unregulated basal activity that results in cAMP synthesis which, in turn, can trigger the expression of lacZ-regulated genes [27] (Figure 1A*a*). Interestingly, the catalytic domain is composed of two independent fragments (T25 and T18) that are not able to re-associate and interact (Figure 1A*b*). However, when both fragments are genetically fused to two proteins of interest, each on different plasmids, the interaction between the two proteins results in functional complementation between the two adenylate cyclase fragments leading to cAMP synthesis (Figure 1A*c*). Functional analysis of *Bordetella pertussis *adenylate cyclase activity can be easily monitored in an *E. coli *strain deficient in endogenous adenylate cylcase (cya^-^) on indicator plates (e.g. LB-X-gal or MacConkey media).

We reasoned that if we constructed a unique plasmid containing both complementary fragments of the reporter enzyme linked by a subcloned human gene of interest, the adenylate cyclase would be functional only in absence of any PSC in the subcloned DNA fragment. In the absence of PSC in the linker, the two domains would be synthesized as a single polypeptide with adenylate cyclase activity (Figure 1B*a*). A PSC in the DNA fragment would stop translation and the second domain required for adenylate cyclase activity would not be present. Any possible translation re-initiation event downstream the PSCs would yield physically unattached fragments that should not be able to re-associate, and hence would not result in a false negative result (Figure 1B*b*).

### The two cya domains expressed as a single polypeptide are catalytically active

**Figure 2:**
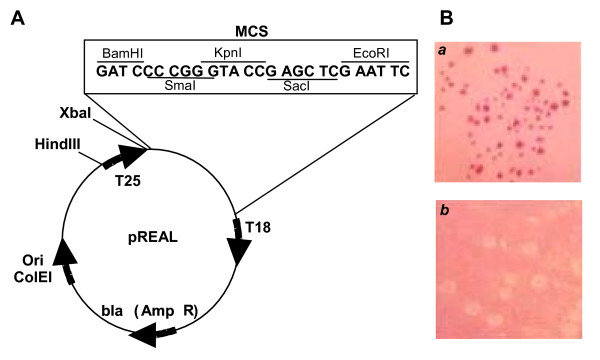
**Design and functional testing of pREAL**. **A**: Design of pREAL with a MSC to insert human DNA fragments. B: Functional testing of pREAL. In *a*, the BTH102 transformants with empty pREAL were plated on MacConkey medium. All the colonies were red after 20 h at 37°C. In *b*, BTH102 transformants with pUT18 were plated on the same medium as control experiment. After 20 h at 37°C, all colonies were white.

To test the hypothesis that the linked domains of the two-hybrid system translated from a single plasmid would have adenylate cyclase activity, we amplified fragment T25 (N-terminal domain of cya) from plasmid pKT25 of the bacterial two-hybrid system, and inserted it upstream and in frame with fragment T18 (C-terminal domain of cya) in plasmid pUT18 to obtain pREAL. Part of the existing MCS present in this plasmid was preserved to facilitate the insertion of human gene fragments (Figure 2A). We transformed an *E. coli *strain (BTH102), deficient in adenylate cyclase, with pREAL and plated the transformants on MacConkey medium. We used as control BTH102 transformed with pUT18. The results revealed that the BTH102 transformed with the pREAL expressed adenylate cyclase activity (100% of the colonies showed an unmistakable red phenotype after 20 h, Figure 2B*a*). In contrast, control plates, transformed with pUT18 that expressed only the C-terminal domain of cya, showed 100% white colonies (Figure 2B*b*). This result confirmed the correct in-frame insertion of T25 upstream T18 and the functional catalytic activity of both domains attached by a short fragment of 10 amino acids.

### The system can accurately detect PSC in human DNA fragments

To test whether pREAL could be used to detect PSCs in human genes, three different fragments of exon 11 of the *brca*1 gene were amplified from a healthy donor. Reported pathogenic stop mutations related to breast and ovarian cancer were introduced by a mutagenic PCR strategy using the wild type DNA as template [28]. A nonsense (NS) and a frameshift (FS) mutation were generated introducing the alterations with two different forward primers. A wild type (WT) version was used as control. All forward primers had BamHI site overhangs and the reverse primer had a 5' EcoRI site to facilitate insertion in the MCS of pREAL. The annealing site of the reverse primer was carefully selected to include ATG and GTG codons post PSC and in-frame with T18, in order to detect translation re-initiation events.

**Figure 3:**
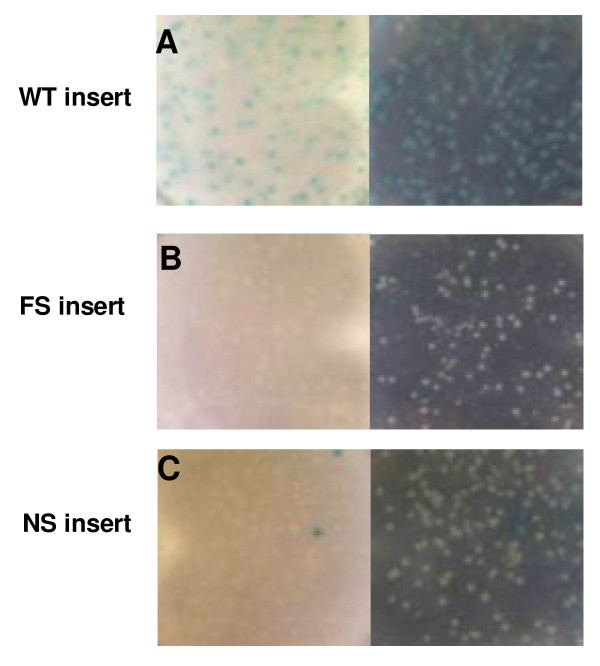
**Detection of inserts with PSCs through blue/white phenotypes in transformed BTH102 bacteria. **Three different fragments, Wild Type (WT), Nonsense (NS), and Frameshift (FS), were obtained by mutagenic PCR from exon 11 of the human *brca1 *gene and inserted in pREAL. BTH102 transformed with pREAL were plated on LB-Xgal medium. All plates are shown on white and black backgrounds. In **A**, BTH102 were transformed with pREAL/WT. Colonies show the blue phenotype consistent with an active adenylate cyclase and a PSC-free DNA insert. In **B**, BTH102 were transformed with pREAL/FS, and in **C**, BTH102 were transformed with pREAL/NS. In the two latter cases, almost all colonies are white, indicating the absence of adenylate cyclase activity, and consistent with the presence of a PSC in the inserted DNA. In C we observed a few Lac^+ ^colonies. A possible source of these false negative results is bacterial phenotype reversion due to endogenous mutations. Notice that this reversion rate does not disturb the diagnosis.

After inserting the different PCR products in pREAL, the BTH102 cells were transformed and plated on LB-X-gal medium. As expected, all bacteria carrying the wild-type insert showed a blue phenotype (Figure 3A) whereas the bacteria carrying the inserts with PSC (frameshift product or nonsense product) showed a white phenotype (Figure 3B and 3C). The experiments showed consistent results either on MacConkey (data not shown) or on LB-Xgal media, confirming that the insertion of a 377–383 bp human sequence allows the functional interaction of fragment T25 with fragment T18. The results also show that any PSC in the DNA linker (by nonsense codon or frameshift) inhibits the catalytic activity of adenylate cyclase.

**Figure 4:**
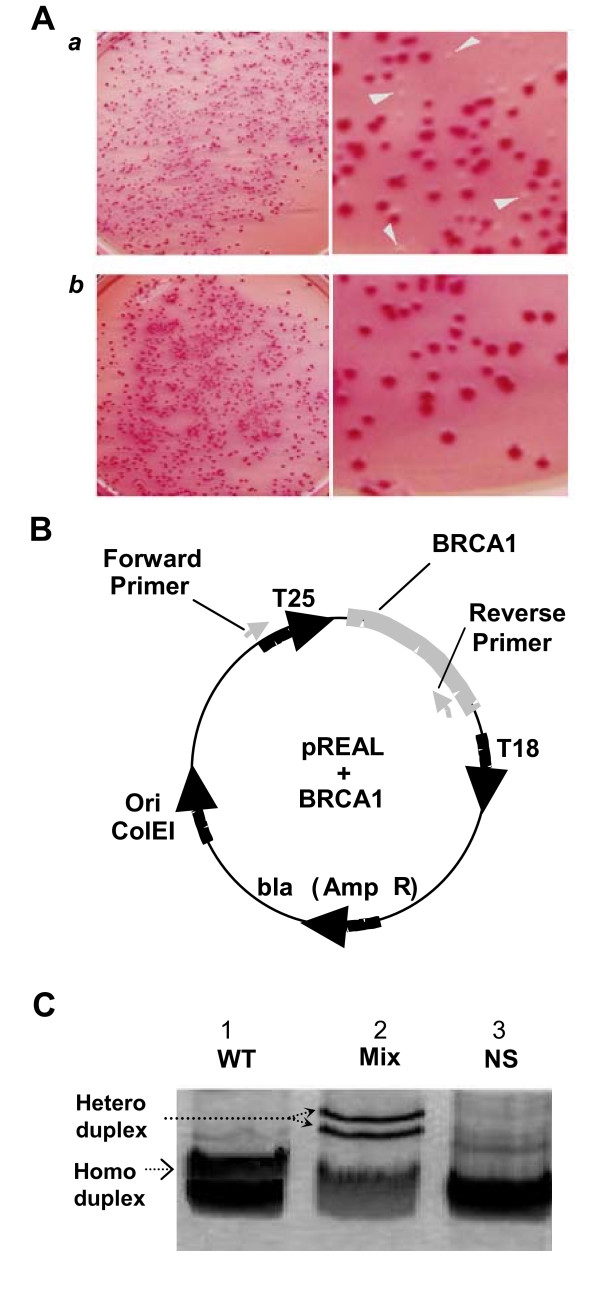
**Detection of heterozygous condition. A**: *a*. BTH102 were transformed with pREAL/WT:pREAL/NS in a 1:1 proportion to test the detection system for heterozygous PCR product. Gray arrows show white colonies. *b*: BTH102 were transformed with pREAL/WT as homozygous control. **B: **localization of the primers (arrows in gray) used to amplify DNA fragments from red and white colonies to detect heteroduplex formation. **C: **DNA was amplified directly from colonies. Red and white colonies were touched with a pipette tip, added to the same PCR mix for amplification, afterwards heat denatured, and allowed to re-hybridize at low temperature. The obtained products were run on a non-denaturing 8% PAGE. In lane 2, the appearance of two slowly migrating bands indicates the presence of heteroduplexes (black arrows) in the mixed sample (WT/NS product). Lanes 1 and 3 are non-combined red and white colonies amplified in different PCR mix, used as controls. In these lanes, the heteroduplex bands are absent.

Most of the PSC mutations in tumor suppressor and mismatch repair genes causing hereditary diseases are present in a single allele. Therefore, any diagnosis system should be able to detect these mutations in heterozygous patients. To mimic a heterozygous PCR product, we mixed in a 1:1 proportion pREAL/wildtype and pREAL/nonsense and transformed the BTH102 with this mix. When plated on MacConkey, the phenotypes showed an equal proportion of white (9.6 colonies/cm^2 ^= 49.7%) and red (9.75 colonies/cm^2 ^= 50.3%) (Figure 4A*a*), whereas the control plates of BTH102 transformed with pREAL/wildtype showed 100% red colonies (Figure 4A*b*).

To confirm that the inserts of the white colonies had different sequences from those of the red colonies, we amplified DNA directly from the colonies (primers are represented with gray arrows in Figure 4B) touching with a single pipette tip a red and a white colony. The resulting mixed PCR product was heat denatured, and allowed to re-hybridize at low temperature. Finally, the mixed DNA product was ran in a non-denaturing PAGE. We expected the formation of homoduplexes (double-strand DNA composed by two complementary strands) in case of a mix with identical sequences, and the detection of heteroduplexes (double-strand DNA composed by two not completely complementary strands) in case of a mix with different sequences. The heteroduplexes are easily detectable by their delayed mobility on an electrophoresis assay. The PAGE showed the formation of heteroduplexes only in the mixed product, confirming that it contained different sequences (Figure 4C). In conclusion, these experiments indicate that insertion of a PSC-containing DNA in pREAL is easily detected by bacterial colony phenotype and that the assay is suitable for analyzing heterozygous samples.

### PSCs were accurately detected even in the presence of re-initiation events

We speculated that the developed diagnosis system would be resistant to translation re-initiation, a common event in bacteria. To detect whether translation re-initiation events were occurring in our system, we used a murine anti-adenylate cyclase monoclonal antibody that binds to the distal portion – amino acids 373–399 – of fragment T18 (C-terminus domain of the protein). Our hypothesis was that, in the absence of PSC, a large product (59 kDa) would be detected by the antibody, whereas in the presence of PSC, if translation re-initiations occurred, smaller products recognized by the antibody should appear.

**Figure 5:**
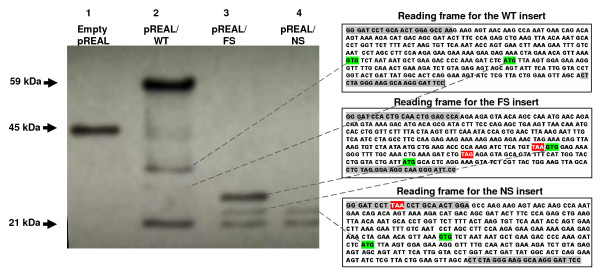
**Detection of re-initiation events by Western Blot. **BTH102 were transformed with empty pREAL, or pREAL ligated to the WildType, Nonsense or Frameshift fragments of the *brca1 *gene. Transformants were cultured, centrifuged, and the protein pellets were resolved on 10% SDS-PAGE. The membrane with the transferred proteins was probed with a monoclonal antibody for fragment T18. The corresponding insert sequences are shown at the right. PCR primer sequences are shown in gray, stop codons in red, and translation initiation codons in green. Lane1: BTH102 transformed with empty pREAL. A 45 kDa band is observed corresponding to the fused T25 and T18 fragments. Lane 2: BTH102 transformed with pREAL/WT (wild type exon 11 of *brca*1). The upper band of 59kDa corresponds to the T25-insert-T18 protein. Two smaller fragments can be observed, likely produced by translation re-initiation events from two downstream and in-frame ATG codons. The lowest band (21 kDa) corresponds to translation re-initiation of fragment T18. Lane 3: BTH102 transformed with pREAL/FS (exon 11 of *brca*1 with a frame shift mutation that introduces a PSC). Notice the absence of the complete 59 kDa fragment due to the presence of a TAA stop codon. The presence of two smaller fragments can be explained by two in frame initiation codons (GTG and ATG). The lowest band corresponds to translation re-initiation of fragment T18. Lane 4: BTH102 transformed with pREAL/NS (exon 11 of *brca*1 carrying a nonsense mutation). The absence of the full-length 59 kDa fragment is caused by the TAA codon included in the primer sequence. One smaller fragment is observed, consistent with an initiation ATG codon. Again, the lowest band corresponds to fragment T18.

Western Blot assays were performed on extracts from BTH102 transfected with empty pREAL, pREAL/WT, pREAL/FS and pREAL/NS. The results in Figure 5 show that small fragments recognized by the antibody were present in all samples, indicating that translation initiation downstream the stop codon was frequent and that it occurred even in the DNA fragment lacking PSC. A careful analysis of the sizes showed that they were consistent with the ATG and GTG codons present in the linker. However, it is worth noting that the full-length product was only present in the adenylate cyclase positive (i.e., red colony) bacteria. The results show that translation re-initiation is a frequent event under the conditions of the assay and that a reporter gene located downstream the inserted DNA would not be a good method to detect PSCs. This observation stresses the importance of the pREAL two-domain strategy developed to detect stop codons in the inserted DNA.

### The system correctly detects a truncating mutation in a HNPCC patient

The pREAL plasmid has proved to be very efficient to detect stop codons introduced in exon 11 of the *brca*1 gene of a wild type DNA, even in heterozygous samples. We wanted to test the system with another gene and in a more realistic situation. For this propose, exon 13 of the *msh*2 gene was amplified from DNA obtained from peripheral blood of a patient from a HNPCC family. This family presents a mutation (TGA>TAA) on codon 711 of this gene. The PCR product was inserted in frame in the MCS of pREAL. Note that the patient was heterozygous; hence, the PCR product contained both, the mutated and the wild type alleles. DNA of an unaffected member of the HNPCC family was used as control.

**Figure 6:**
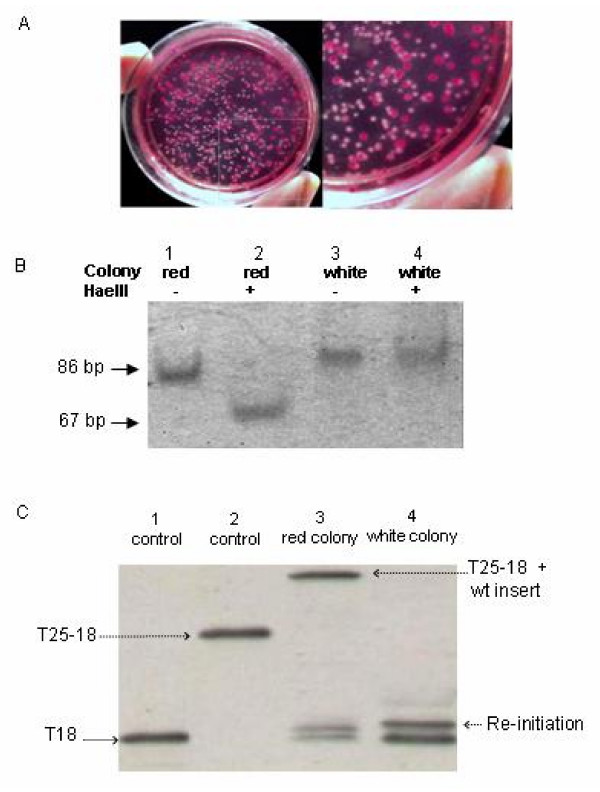
**Application of the method to a HNPCC patient with a PSC. A**: BTH102 were transformed with pREAL carrying a *msh*2 insert obtained from a heterozygous patient. The proportion of red/white colonies was 51.7% and 48.3% respectively. **B**: Confirmation of the genotype of the different colonies by PCR and RFLP resolved on 10% non-denaturing PAGE. Lane 1 shows the 86bp PCR product of a red colony, without enzymatic incubation. Lane 2: PCR product obtained from a red colony, with subsequent enzymatic incubation. The 67 bp band results from the complete cleavage of the wild type allele in two smaller fragments of 67 bp and 19 bp (band not shown). Lane 3: PCR product obtained from a white colony, without enzymatic incubation. Lane 4: PCR product obtained from a white colony, with subsequent enzymatic incubation. The 86bp band in the mutated allele is not recognized by HaeIII and is not cleaved. **C**: Western blot analysis of red and white colonies. In lanes 1 and 2, controls for fragment T18 and fragment T25-18 are shown. In lane 3, the western blot on the red colony reveals the presence of the complete adenylate cyclase, with the wild type version of the *msh*2 fragment. Notice two re-initiation events: one that corresponds to fragment T18, and a larger band, that corresponds to the presence of an ATG codon in the insert. In lane 4, the Western blot of the white colony confirms the absence of full-lenght adenylate cyclase, and reveals the same re-initiation events seen in the red colonies, one corresponding to T18 and another corresponding to an ATG post PSC.

In this instance, transformation of BTH102 was performed directly with the ligation product avoiding a previous miniprep purification step in order to simplify the method. To improve the amount of transformants, the culture expansion time was increased to 3 h. After 20 h of incubation on MacConkey at 37°C, the transformants with the DNA from an unaffected individual showed 12.5 colonies/cm^2^; all of them with a red phenotype (data not shown). In contrast, transformants from the heterozygous patient showed 12.5 white colonies/cm^2 ^(48.3%) and 13.4 red colonies/cm^2 ^(51.7%) (Figure 6A). The results clearly indicate that the method accurately identifies a PSC-containing DNA segment even in the presence of a non-mutated allele. To confirm the genotype of the different colonies, a PCR from red and white colonies and subsequent restriction enzyme incubation was performed. The results shown in Figure 6B confirm the presence of the wild type insert in red colonies (PCR product completely cleaved by the restriction enzyme, Figure 6B, lane 2) and the mutated allele in white colonies (PCR product not recognized and not cleaved by the restriction enzyme, Figure 6B, lane 4).

In the assay with the *brca*1 exon, the reverse primer was designed in order to include ATG and GTG sequences on purpose to test the robustness of the method to translation re-initiation problems. We assessed the occurrence of re-initiation in this more realistic assay with the DNA amplified from a patient. Western Blot assays revealed the presence of re-initiation events in red and white colonies (Figure 6C, lanes 3 and 4), proving that a simpler plasmid, based on the expression of a single reporter would have strong limitations due to re-initiation events. Notice that, as expected, white colonies do not express the full-length (active) adenylate cyclase (Figure 6C, lane 4). These observations show that the pREAL strategy can be used in a relatively simple diagnostic scheme: PCR amplification from genomic DNA from patients, ligation into pREAL, transformation with the ligation product into BTH102, and bacterial phenotype screening in MacConkey or LB-Xgal media.

## Discussion

The diagnosis of hereditary diseases by molecular biology methods has proved to be a powerful tool for the managing of affected families and, in many cases, for the treatment of affected members. With a growing number of genes identified as related to specific syndromes, these methods are of great interest to the medical community. Although feasible, the complete sequencing of the suspected gene is still not efficient. Moreover, many mutations may be normal polymorphisms without pathological significance. Morbid mutations are frequently related to inactivation of the gene product by the conversion of wild type triplets to stop codons, or changes in the reading frame that usually introduce stop codons. Therefore, the detection of PSCs in disease-related genes is usually of great importance for the diagnosis of hereditary diseases.

We describe here a bacterial system that allows the detection of PSCs in human genes by a simple colony phenotype screening. The idea was originated in the two-hybrid system based on a reconstituted signal transduction pathway in bacteria, developed by Karimova et al [26] in the Pasteur Institute. In our design, the in vivo translation of *Bordetella pertussis *adenylate cyclase fragments linked by a PSC-free inserted DNA triggers the expression of cAMP-dependent genes. We took advantage of the fact that the T25 and T18 fragments are unable to recognize each other and cannot reconstitute a functional enzyme when expressed as separate entities. However, if they are attached by a polypeptide chain, the protein is active and cAMP is synthesized. Ultimately, cAMP, upon binding to CAP, activates the transcription of catabolic operons, allowing the bacteria to ferment carbohydrates such as maltose or lactose. We show here that any PSC in the DNA inserted between the adenylate cyclase fragments inhibits the activity of the translation product. We also show that translation re-initiation events do occur and that the pREAL system is resistant to this source of false negative results. Indeed, re-initiation was a very common event observed even in sequences lacking PSCs. We showed by Western blot that most of the possible initiation codons in the inserted fragment were used and generated a detectable polypeptide in the assay.

Patients with hereditary mutations in tumor suppressor genes are always heterozygous, because homozygous individuals are unviable. Hence, a mixed PCR product is obtained when DNA from these patients is amplified. However, a single PCR product is inserted per copy of the pREAL plasmid; in turn, only one pREAL copy transforms a cya^- ^bacterium that expands in a clone on the plate. Therefore, each colony represents one allele. In conclusion, this method allows the analysis of separated alleles of heterozygous patients, avoiding possible allele-interfering events affecting other mutation detection systems [29].

For the application of the pREAL plasmid, we propose a diagnosis algorithm starting from genomic DNA extracted from peripheral blood of index patients suspected of FAP, HNPCC, Familial Breast Cancer, or any other disease related to PSCs. Each exon should be amplified with specific primers carrying endonuclease cleavage sequences. The amplification products should be subsequently inserted into pREAL, and BTH102 cells transformed directly with the ligation product. After plating on MacConkey for 20 hours, heterozygous exons with a PSC in one allele would be detected by a 50/50 red/white phenotype. To test the rest of the family members, only the affected exon should be amplified and inserted into pREAL to detect the presence or absence of the PSC.

The more complex step in this algorithm is the ligation of the PCR product into pREAL because it involves incubation with restriction enzymes and purification of the cleaved insert. Some strategies for directional in frame ligation have been developed by commercial companies (TOPO TA Cloning kits, Invitrogen Life Technologies). In addition, mRNA could be used as template, allowing the amplification by RT-PCR of consecutive exons and therefore reducing the cleavage/ligation steps. However, one of the disadvantages of the RNA-based approaches is the requirement for abundantly expressed and stable transcripts. In particular, mRNAs carrying PSCs are degraded by a specific mechanism (Nonsense Mediated Decay system) that prevents the translation of truncated proteins [30]. Hence, the amount of mRNA carrying PSCs is highly reduced. Therefore, mRNA might not be a recommendable starting material for routine diagnosis.

Another predicted problem with the pREAL strategy is the possibility that the three-dimensional structure of the polypeptide chain linking the adenylate cyclase fragments may interfere with the functional interaction between the domains yielding an inactive enzyme. This false positive result would be easily detected because white colonies will be observed with control wild type inserts. If this problem is detected for an exon of a gene, Western blot analysis should be required to test the presence of full-length protein in the colonies. Alternatively, a different mutation detection system should be used for this exon. All in all, adenylate cyclase activity was detected with the three different linkers tested in the present report (i.e., empty vector, and pREAL carrying wildtype fragments of exon 13 of the msh2 gene and exon 11 of the brca1 gene). In conclusion, before using for clinical diagnosis, this method should be tested for each exon of the gene of interest in control and PTC containing samples. Moreover, the accuracy and efficiency of the system to detect PTCs should be confirmed by future prospective studies.

## Conclusion

The pREAL plasmid is applicable for the detection of PSCs in human genes related to different diseases and is resistant to translation re-initiation events. The diagnosis steps are easy and have a low cost. They require only a laboratory equipped for DNA extraction, PCR amplification, ligation, and bacterial culture. The most relevant biological advantages of the method are the detection of only pathologic mutations, thus avoiding subsequent analysis for possible polymorphisms, and the analysis of separated alleles in heterozygous patients.

For human genes with a high frequency of PSCs, this tool can be applied as a first screening method in an exon by exon strategy. In developing countries, where present methods of molecular diagnoses of hereditary cancers are still expensive and unaffordable for many families, this mutation detection system may become a useful and applicable tool.

## Methods

### Strains and growth media

DMH1 (F^-^, cya-854, recA1, endA1, gyrA96, thi1, hsdR17, spoT1, rfbD1, glnV44), BTH101 (F^-^, cya-99, araD139, galE15, galK16, rpsL1, hsdR2, mcrA1, mcrB1), and BTH102 (F^-^, cya-99, recA1, sr1A::Tn10) are adenylate cyclase deficient strains (cya^-^) and were used for the bacterial phenotype screening setup. XL1-B strain (F^- ^lac, proAB^+ ^lacI^q ^lacZΔM15 Tn10 Tet^r^, cya^+^, recA1 endA1 gyrA96 thi-1 relA1hsdR17 supE44) was used to clone the original plasmids of the two hybrid system and the recombinant plasmid pREAL. Frozen competent cells were thawed and transformed using a CaCl_2 _protocol. The growth medium used was rich Luria-Bertani (LB) supplemented when required with 100 μg/ml ampicillin or 50 μg/ml kanamycin. Screening for the ability to ferment lactose was performed either on MacConkey agar plates without additional maltose or on LB plates containing 40 mg/ml X-gal (5-bromo-4-chloro-3-indolyl-β-D-galatopyranoside).

### Development of the pREAL plasmid

Two plasmids from the bacterial two-hybrid system were used: pKT25 (coding for the T25 fragment – aminoacids 1–224 – of the catalytic domain of adenylate cyclase) and pUT18 (coding for the T18 fragment – aminoacids 225–399 – of the catalytic domain of adenylate cyclase). To generate pREAL, we inserted the T25 sequence in pUT18, upstream and in frame with the T18 fragment. Cloning attempts with pREAL in cya^- ^strains (DMH1, BTH101) were not successful and surprisingly the best cloning results were obtained in XL1-B bacteria (cya^+^) that seemed to resist the hypothetical excess of cAMP. The effect of pREAL on bacterial phenotype was better observed in BTH102, a rapid growing, cya^-^, endA, but also recA1 strain. Competent BTH102 (50 μl) were transformed with 2 μl of pREAL and after an expansion at 37°C for 45 min, 15 μl were plated on MacConkey medium with 100 μg/ml ampicillin. Addition of 1% maltose or 0.1 mM IPTG to the medium did not significantly improve the colony number or growth rate.

### Generation of human DNA fragments with PSCs by mutagenic PCR and insertion in the pREAL

Three versions of the exon 11 of the human *brca*1 gene were generated with three different forward primers: i) "nonsense product"(NS), with a TAA nonsense codon at position 672 of the gene (5' GG GAT *CCT ****TAA ***CCT GCA ACT GGA), ii) "frame-shift product"(FS), with an insertion of two base pairs, which provoked a frame shift and a PSC at position 698, 25 codons downstream of the insertion site (5' GG GAT CC***A C***TG CAA CTG GAG CCA A), and iii) "wild type product"(WT) (5'GG GAT CCT GCA ACT GGA GCC AA). Each of the forward primers was combined with the same reverse primer (5' GGAATTCCTTGCCTTCCCTAGAG) to generate PCR products of 383, 379, and 377 bp, respectively. The annealing site for the reverse primer was selected to include translation initiation codons (ATG and GTG) downstream of the PSCs. The three different amplified products were cut with EcoRI and BamHI (1 h at 37°C), purified from an agarose gel, and inserted in the MCS of pREAL. After transforming XL1-B, the plasmids were isolated using the Wizard Plus Miniprep Purification system (Promega Corporation, Biodynamics, Buenos Aires, Argentina). BTH102 were transformed with pREAL/WT, pREAL/FS, pREAL/NS, and with a 1:1 mix of pREAL/WT and pREAL/NS. After 45 minutes of expansion, cultures were 1:3 diluted with LB and 15 μl were plated on MacConkey medium. Without this dilution, we observed a few Lac^+ ^colonies in BTH102/PSC plates. A possible source of these false negative results is bacterial phenotype reversion due to endogenous mutations. The informed frequency of this events is 10^-7 ^– 10^-8 ^[26]; however we observed a higher reversion rate (3–6%), likely because of natural selection acting on the cya^- ^bacteria. Notice that this reversion percentage would not disturb the diagnosis. Reverted colonies were seldom found when the total number of bacteria per plate was reduced by a 1:3 dilution with LB.

### Heteroduplex analysis from red and white colonies

To confirm that the different colored colonies had different inserts, white and red clones from the plate were touched with a pipette tip. Both tips were added to a same PCR mix to perform a single amplification from both colonies using the forward primer (5' GGTCCAAGCTTGGCTATGACCATGCAG) previously used to amplify fragment T25, and the reverse primer used for *brca*1 exon 11. Then, the PCR products were boiled 5 min at 95°C and renatured at RT for 30 min to allow the formation of heteroduplexes. The samples were run on a non-denaturing 8% PAGE for 3 h at 120 Volts. As control, separately PCR products from white and red colonies were used. PCR products were visualized by ethidium bromide staining and UV light exposure of the gel.

### Western blot of T18 to detect re-initiation events

50 μl of bacterial cultures were centrifuged at 10,000 rpm. The bacterial pellets were resuspended in sample buffer (10 μl), boiled for 5 min, and resolved on 10% SDS-PAGE at 15mA. The gels were electroblotted onto nitrocellulose. Membranes with transferred proteins were incubated with murine anti-adenylate cyclase toxin monoclonal antibody (3D1, List Biological Laboratories, INC, Campbell, California, USA) (0.16 μg/ml) as primary antibody, followed by incubation with anti-mouse IgG coupled to HRP (Sigma-Aldrich, St Louis, MO, USA) (0.25 μg/ml) as secondary antibody. Chemiluminescence reaction using the ECL kit (Amersham Bioscience, Buenos Aires, Argentina) was carried out for 1 min followed by exposure to Kodak XAR Radiograph film (Eastman Kodak, Rochester, NY, USA) for 15 and 60 sec.

### Application for the detection of a truncating mutation in a HNPCC patient

DNA of a previously diagnosed HNPCC patient [31], heterozygous for a PSC, was used as template. This patient has a CGA>TGA nonsense mutation in codon 711 of gene *msh*2. Primers were designed to include the PSC with 5' overhangs for EcoRI and BamHI sites. The sequences of the primers were: forward 5' AGGACTAAGGATCCATTTAT and reverse 5' TATGGAATTCCAAGCAGTTTCCAACAT. A 277 bp PCR product was obtained, incubated with EcoRI and BamHI for 1 h at 37°C, purified from an agarose gel and inserted in the MCS of pREAL. To avoid the subsequent miniprep purification step and carry out directly the diagnosis screening, it was necessary to increase the concentration of transformants before plating. Adding glucose during the expansion step and removing it before plating did not give a different number of colonies on MacConkey or LB-Xgal. However, a three-fold longer expansion time of the transformants before plating produced enough colonies (80–100/plate) for a heterozygous diagnosis. Hence, 50 μl of BTH102 were transformed with 2 μl of recombinant plasmid, expanded in LB in a shaker at 37°C for 3 hours. The culture was centrifuged and the pellet was plated on MacConkey medium and incubated for 20 hours at 37°C.

To evaluate the different bacterial phenotypes on the plate of the heterozygous patient, Western Blot assays were performed as described on white and red colonies. To confirm the presence of the PSC in white colonies and its absence in red colonies, a previously engineered PCR-based diagnosis method was used [31]. The strategy is to introduce a mutation that abuts the PSC site with the forward primer. This mutation creates a restriction site for the HaeIII enzyme only in the wild type allele, but not in the allele carrying the PSC. Sequences of the diagnosis primers were: forward, 5'TTGTGGACTGCATCTTAGGC and reverse, 5' CAGTTTCCAACATTTCAGCCA. White and red colonies from the plate were touched with a pipette tip, and each tip was separately added to a PCR mix with the mentioned primers to run the amplification. PCR products were then incubated with the restriction enzyme HaeIII for 1 h at 37°C, and run on non-denaturing 10% PAGE.

## Authors' contributions

SR worked on the design and construction of pREAL, the transformation of cya^- ^strains, and the Western Blots assays. DM worked on the design and insertion of the *brca*1 and *msh*2 sequences, followed by the respective cya- transformation, Western Blots, and sequence analysis. He also collaborated in the draft of the manuscript. LG was involved in experimental support. LM advised on concept design, on the experimental work, and on the critical revision of the manuscript. MR developed the concept and designs of the method, guided the project and drafted the manuscript.
